# Identification of male heterogametic sex‐determining regions on the Atlantic herring *Clupea harengus* genome

**DOI:** 10.1111/jfb.14349

**Published:** 2020-05-22

**Authors:** Sunnvør í Kongsstovu, Hans Atli Dahl, Hannes Gislason, Eydna Homrum, Jan Arge Jacobsen, Paul Flicek, Svein‐Ole Mikalsen

**Affiliations:** ^1^ Amplexa Genetics A/S Tórshavn Faroe Islands; ^2^ Faculty of Science and Technology University of the Faroe Islands Tórshavn Faroe Islands; ^3^ European Molecular Biology Laboratory European Bioinformatics Institute Cambridge UK; ^4^ Faroe Marine Research Institute Tórshavn Faroe Islands

**Keywords:** Atlantic herring, *Clupea harengus*, genome‐wide association study, sex determination

## Abstract

The sex determination system of Atlantic herring *Clupea harengus* L., a commercially important fish, was investigated. Low coverage whole‐genome sequencing of 48 females and 55 males and a genome‐wide association study revealed two regions on chromosomes 8 and 21 associated with sex. The genotyping data of the single nucleotide polymorphisms associated with sex showed that 99.4% of the available female genotypes were homozygous, whereas 68.6% of the available male genotypes were heterozygous. This is close to the theoretical expectation of homo/heterozygous distribution at low sequencing coverage when the males are factually heterozygous. This suggested a male heterogametic sex determination system in *C. harengus*, consistent with other species within the Clupeiformes group. There were 76 protein coding genes on the sex regions but none of these genes were previously reported master sex regulation genes, or obviously related to sex determination. However, many of these genes are expressed in testis or ovary in other species, but the exact genes controlling sex determination in *C. harengus* could not be identified.

## 1 INTRODUCTION

The evolution of sexual reproduction has resulted in several sex determination systems, with gonochorous organisms (the stable separation of sexes in different individuals), stable hermaphrodites and organisms that change sex dependent on age, environmental and/or social cues (Devlin & Nagahama, 2002; Shen & Wang, 2014). Each of the different systems has evolved independently several times through evolutionary history, and even within each system there might exist several mechanisms for determining the sex of an organism (Ashman *et al.*, 2014). The best‐known system is the XY sex chromosomes found in mammals, where the females have two X chromosomes and the males have an X and a Y chromosome. Thus, the XY system is a male heterogametic system. The sex‐determining region Y (*SRY*) gene is located on the Y chromosome and signals to the body to develop into a male rather than a female, which is the default (Kashimada & Koopman, 2010). The ZW system is a similar system where the females are heterogametic. This system is found in birds and some amphibians (Bull, 1983; Yoshimoto & Ito, 2011). These two systems do not represent the complexity of sex determination systems in the animal kingdom. Systems with only one sex chromosome also exist, for example the X0 system where males have only one sex chromosome and the Z0 system where females have only one (Bachtrog *et al.*, 2014; Clinton, 1998). Furthermore, sex determination systems can be more complex, with multiple chromosomes or genes affecting the sex (Bachtrog *et al.*, 2014; Roberts *et al.*, 2016). There are even systems where age and size (Allsop & West, 2003), societal factors (Buston, 2003; Fricke, 1979) or environmental factors such as temperature (Pieau, 1996) play crucial roles in sex determination. In some organisms, both genetic and environmental factors are involved in determining the sex, for example in the Nile tilapia *Oreochromis niloticus* (Linnaeus 1758) (Baroiller *et al.*, 2009) and Atlantic silverside *Menidia menidia* (Linnaeus 1766) (Lagomarsino & Conover, 1993).

Teleost fish display a variety of sex determination systems (Brykov, 2014; Devlin & Nagahama, 2002) and the plasticity of teleost genomes makes it possible for new systems to evolve relatively quickly. This makes teleost fish good candidates for studying the evolution of sex determination. Although there are sex determination systems in fish that are influenced by nongenetic factors (see the references above), genetic sex determination seems to be more common. The male heterogametic system (hereinafter ‘the XY system’) has been established in some fish species, for example bighead carp *Hypophthalmichthys nobilis* (Richardson 1845) and silver carp *Hypophthalmichthys molitrix* (Valenciennes 1844) (Liu *et al.*, 2018), as has the female heterogametic system (hereinafter ‘the ZW system’) in half‐smooth tongue sole *Cynoglossus semilaevis* Günther 1873 (Chen *et al.*, 2014). The cichlid fishes of Lake Malawi have families with the XY system and others with the ZW system, but notably the species *Metriaclima pyrsonotus* (Stauffer, Bowers, Kellogg & McKaye 1997) has both these systems, showing strong epistatic interactions between them (Ser *et al.*, 2010). Several polygenic systems are also found in fish, such as the European sea bass *Dicentrarchus labrax* (Linnaeus 1758) (Palaiokostas *et al.*, 2015) and the cichlid fish *Astatotilapia burtoni* (Günther 1894) (Roberts *et al.*, 2016). There are even individuals from the same species that have different sex determination systems, for example some zebrafish *Danio rerio* (Hamilton 1822) laboratory strains have lost the sex‐determining region that is present in wild‐type *D. rerio*, and therefore have evolved a new polygenic system that is still not fully understood (Wilson *et al.*, 2014).

In some organisms (*e.g*., mammals and birds), sex chromosomes have evolved that contain master sex regulation (MSR) genes that control the sex of the organism, as with the previously mentioned *SRY*. Most species of fish do not have specific heteromorphic chromosomes that control sex, but have regions on autosomes that are associated with sex determination. These sex regions sometimes contain MSR genes or candidate MSR genes, such as the Y chromosome‐specific anti‐Müllerian hormone (*amhy*) gene in Patagonian pejerrey *Odontesthes hatcheri* (Eigenmann 1909) (Hattori *et al.*, 2012) or the sexually dimorphic gene on the Y chromosome (*sdy)* in rainbow trout *Oncorhynchus mykiss* (Walbaum 1792) (Yano *et al.*, 2012). However, sometimes no obvious causal genes are found on these regions that have been associated with sex, such as in the mandarin fish *Siniperca chuatsi* (Basilewsky 1855) (Sun *et al.*, 2017).

In the Clupeidae family, few species have been studied regarding their sex determination systems. In the Tree of Sex Consortium database (Ashman *et al.*, 2014), only six Clupeiformes species are mentioned; four of these are a part of the Clupeidae family. Two are hemaphrodites [the toli shad *Tenualosa toli* (Valenciennes 1847) and the longtail shad *T. macrura* (Bleeker 1852)], whereas the Argentine menhaden *Brevoortia pectinata* (Jenyns 1842) and Brazilian menhaden *B. aurea* (Spix & Agassiz 1829) are both gonochoristic. *B. pectinata* is homomorphic and *B. aurea* is male heterogametic with X_1_X_2_Y sex chromosomes (Brum, 1992). In addition, the Gulf menhaden *Brevoortia patronus* Goode 1878, the yellowfin menhaden *Brevoortia smithi* Hildebrand 1941 and the Atlantic menhaden *Brevoortia tyrannus* (Latrobe 1802) are gonochoristic and homomorphic (Doucette Jr & Fitzsimons, 1988), but their sex determination systems are not known.

The sex determination system of the commercially important Atlantic herring *Clupea harengus* L. has not yet been described. Increasing the knowledge of sex determination at this branch of the tree of life would further elucidate the evolution of sex determination in teleost fish. We therefore undertook this study to find regions on the *C. harengus* genome that are associated with sex determination.

## 2 MATERIALS AND METHODS

### 2.1 Ethical statement

The *C. harengus* samples were received from stock assessment cruises and commercial catches in the north‐east Atlantic. No fish were caught or handled while alive for the purpose of this project. All fish were dead when they were selected for the study. Thus, the research did not involve animal experimentation or harm, and required no ethical permits.

### 2.2 Samples and DNA extraction

Kidney samples were taken from 103 adult Atlantic herring, originated from four stocks, with ages ranging from 3 to 12 years with an average of 6.1 years. The sex was determined by visual inspection of the gonads by experienced staff at the Faroe Marine Research Institute, revealing 48 females and 55 males. DNA was extracted from the kidney tissue of these fish using an AS1000 Maxwell 16 instrument (Promega, Madison, WI, U.S.A.) and the Maxwell 16 Tissue DNA Purification Kit (Promega). DNA concentrations were measured using a Qubit 3.0 fluorometer (ThermoFisher Scientific,Waltham, MA, U.S.A.).

### 2.3 Sequencing

The isolated DNA from each individual was fragmented to roughly 300 bp using a Covaris M220 focused‐ultrasonicator (Covaris, Chicago, IL, U.S.A.), and the libraries were prepared using the KAPA LTP Library Preparation Kit Illumina Platforms (KAPABiosystems, Wilmington, MA, U.S.A.). Approximately 1 μg of input DNA was used for each library and a final concentration of 1 μM of the 6 bp adapters (Pentabase, Odense, Denmark). After the ligation step, double‐sided size selection for fragments between 250 and 450 bp was performed, following the manufacturer's instructions. The libraries were amplified for two to four cycles, depending on post‐ligation concentration, and no further size selection was performed.

The finished libraries were quantified using the KAPA Library Quantification Kit (KAPABiosystems), as per the manufacturer's instructions. After quantification, the libraries were pooled to equal proportions and paired‐end sequencing was carried out on a NextSeq500 benchtop sequencer (Illumina, San Diego, CA, U.S.A.) using the High Output v2 Kit (Illumina) for 151 cycles.

### 2.4 Data processing and variant calling

Trimmomatic v0.36 was used to remove adapter sequences and trim low‐quality bases with an average quality score lower than 20 (sliding window of four bases) from the paired‐end data (Bolger *et al.*, 2014). AfterQC v0.4.0 was used to remove the polyG reads (Sun *et al.*, 2017) and FastQC v0.11.5 was used to assess the quality of all the sequencing data before and after adapter removal and low‐quality base trimming (Andrews, 2010). The sequencing reads are available in the European Nucleotide Archive repository, with accession numbers from ERS4329014 to ERS4329116.

The data were then aligned to the *C. harengus* chromosome‐level genome assembly (GCA_900700415.1_Ch_v2.0.2) using BWA‐MEM v0.7.15 with default parameters (Li, 2013), and SAMtools v1.3 was used for sorting, converting (between SAM and BAM file formats) and removing PCR duplicates from the alignment files (Li *et al.*, 2009). Single nucleotide polymorphisms (SNPs) were called using FreeBayes v1.1.0 (Garrison & Marth, 2012), and SNPs not in the Hardy–Weinberg equilibrium, SNPs with a minor allele frequency lower than 0.01, SNPs with a quality score lower than 20 (QUAL <20) and SNPs with coverage lower than 2 (DP < 2) were filtered out. However, data with coverage 1 were used when comparing the experimental genotype data with a theoretical model of how to infer reliable diploid genotypes from sequence data (see below).

Sex‐specific insertions and deletions (indels) on the regions associated with sex were also called using FreeBayes and the aligned sequencing data from males and females separately. Indels with more than 30% of the genotypes missing (due to low coverage) and indels where homozygous reference allele genotypes were present were filtered out.

### 2.5 Association analysis

A genome‐wide association study (GWAS) was performed using Plink v1.07 (Purcell *et al.*, 2007) to test whether any of the SNPs identified were associated with sex. For the GWAS, phenotypic sex was used as cases (females) and controls (males), and the Cochran–Mantel–Haenszel test was used to account for the population stratification (−mh option in Plink). The Bonferroni correction for multiple testing assumes that each test is independent. This assumption is not always true for a GWAS because of linked SNPs, thus the Bonferroni correction can be considered too conservative. In human genetics, the genome‐wide significance *P* value threshold of *P* = 5 × 10^−8^ is standard for common‐variant GWAS (Fadista *et al.*, 2016). In this case, the null hypothesis is rejected if *P* < 5 × 10^−8^ or –log_10_(*P*) > 7.3. However, no studies have been published that show this value to be true for herring. Therefore, we chose to use the conservative Bonferroni correction (0.05/number of tests, which was 7,614,270) *P* = 0.66 × 10^−8^ and reject the null hypothesis of no association for –log_10_(*P*) > 8.2. The R packages qqman (Turner, 2014) and ABHgenotypeR (Furuta *et al.*, 2017) were used for visualization of the results.

### 2.6 Statistical analysis

We compared the experimental genotype data with a theoretical model of how to infer reliable diploid genotypes from the sequence data. In this model, the probability of having *x* identical reads given homozygous genotypes is *P*(*x*|*hom*) = 1, while the probability of having *x* identical reads given heterozygous genotypes is *P*(*x*|*het*) = 1/2^*x*− 1^ (Chenuil, 2012). For the number of identical reads (*x*) ranging from 1 to 21, we plotted the predicted proportions of homozygous genotypes together with the experimental data. The error bars of the experimental data correspond to the 95% confidence interval of the exact binomial test in R. We made no comparison for the number of identical reads higher than 21 because of a low number of samples with such high read coverage.

### 2.7 Search for causal genes

Possible orthologs for the genes in the significant regions were found *via* OrthoDB (Kriventseva *et al.*, 2019). If nothing was found, a blast search of the gene sequence was performed to identify potential orthologs. The functions of the orthologs were investigated in the literature as well as in the UniProt database. The R/Bioconductor package VariantAnnotation (Obenchain *et al.*, 2014) was used together with the Ensembl annotation of the genome (*C. harengus*.Ch_v2.0.2.98) to investigate whether the SNPs identified in this study were located in intergenic regions, promoters, exons or introns.

Furthermore, the sequences of 17 known sex determination or differentiation genes in fish were blasted against the *C. harengus* genome to investigate if any of these genes were present but not predicted for the *C. harengus* genome. The FASTA sequences were obtained from public repositories and blasted against the *C. harengus* genome using BLAST+ (Camacho *et al.*, 2009) with default parameters. These genes are listed in Supporting information Table S1.

## 3 RESULTS

### 3.1 Identification of sex regions on the *C. harengus* genome

**FIGURE 1:**
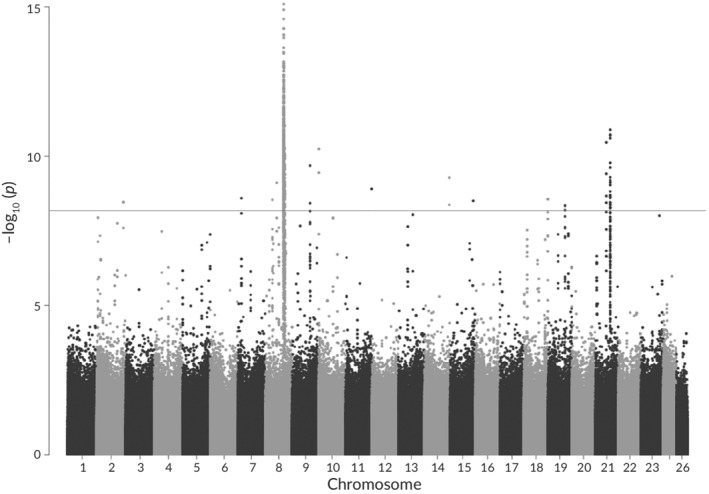
Manhattan plot showing –log of the *P* values from the GWAS investigating sex determination regions on the Atlantic herring *Clupea harengus* genome. The horizontal line indicates the genome‐wide significance threshold [−log_10_(*P*) = 8.2]

SNPs were found *via* low‐coverage whole‐genome sequencing, and a GWAS was conducted to identify the regions on the genome associated with sex, similar to Purcell *et al*. (2018). Whole‐genome sequencing of 103 *C. harengus* (48 females and 55 males) resulted in 267× coverage of the *C. harengus* genome (122× coverage of the female genome and 144× coverage of the male genome; Table 1). After SNP calling and filtering, 7,614,270 SNPs were identified. A GWAS was performed to find genomic regions associated with sex, resulting in 552 SNPs significantly associated with sex. Potentially spurious findings were filtered out based on their relatively poor *P* values and no other significant *P* values in close proximity (Reed *et al.*, 2015). The remaining 529 SNPs associated with sex (hereinafter referred to as the sex SNPs) aggregated on chromosomes 8 and 21 (Table 2 and Figure 1) and are listed in Supporting Information Table S2.

**TABLE 1:** Number of reads generated by low coverage sequencing and coverage of the 850 Mb Atlantic herring *C. harengus* genome

	No. of reads				Coverage			
	Pre QC	$\;\bar{X}$	Post QC	$\;\bar{X}$	Pre QC	$\;\bar{X}$	Post QC	$\;\bar{X}$
Total	2,094,755,946	19,577,158.4	1,549,740,080	14,483,552.2	394.3	3.8	267.1	2.6
Female	945,494,924	19,295,814.8	708,052,772	14,450,056.6	178.0	3.7	122.5	2.6
Male	1,149,261,022	20,162,474.1	841,687,308	14,766,444.0	216.3	3.9	144.7	2.6

*Note:* Quality control consisted of trimming of low‐quality sequences and adapter sequences (see method). QC, quality control; $\;\bar{X}$, average per individual.

**TABLE 2:** Regions of the Atlantic herring *C. harengus* genome and number of SNPs associated with sex that were identified in the GWAS

Chromosome	Position	No. of SNPs
8	21,063,400–22,268,779	488
21	17,047,390–17,055,230	41

**FIGURE 2:**
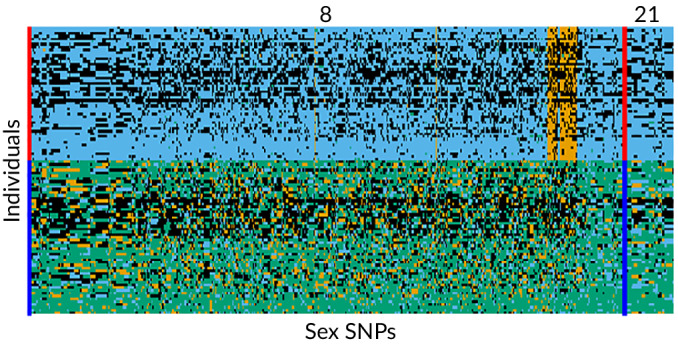
Genotypes for the SNPs significantly associated with sex in Atlantic herring *Clupea harengus*. The dark blue and red vertical lines represent male and female individuals, respectively. The homozygous reference allele genotypes are light blue (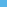). The homozygous alternative allele genotypes are orange (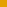). The heterozygous genotypes are green (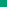). No genotyping data available is black (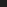)

Investigation of the sex SNPs showed that 17,161 out of the 17,267 available female genotypes were homozygous, whereas 14,639 out of the 21,333 available male genotypes were heterozygous (Figure 2 and Table 3). A closer look at the SNP with the lowest *P* value (Chr8:21,120,262, *P* = 8.058 × 10^–16^) showed the general genotype pattern. Thirty‐eight females had genotyping data for this SNP and all of them were homozygous for the reference allele. Forty‐two males had genotyping data for this SNP, one was homozygous for the reference allele, nine were homozygous for the alternative allele and 32 were heterozygous. All the sex SNPs showed a similar pattern where the majority of females are homozygous and the majority of males are heterozygous (Table 3). This suggested a male heterogametic or XY sex determination system for *C. harengus*.

**TABLE 3:** Genotype count for the 529 SNPs associated with sex in Atlantic herring *C. harengus*

Genotype	Females	Males	Total
Homozygous (reference + alternative)	17,161 (16,418 + 743)	6694 (3522 + 3172)	23,855
Heterozygous	106	14,639	14,745
Total	17,267	21,333	38,600

The erroneous call at low sequence coverage of homozygotes from factual heterozygotes is as expected, and was theoretically investigated in a previous study (Chenuil, 2012). Thus, the true rate of heterozygotes in our data was higher than our result of 68.6%, but this could not be detected due to low sequencing coverage, resulting in male genotypes possibly being wrongly called as homozygous. Table 4 shows the average coverage of homozygous and heterozygous genotypes, and the average coverage of the heterozygous male SNPs was higher than the average coverage for the homozygous male SNPs. This indicated that some of the homozygous genotypes could be wrongly called due to low coverage.

**TABLE 4:** Average coverage for the individual SNPs associated with sex in Atlantic herring *C. harengus*

Genotype	Females			Males		
	Average	S.D.	*n*	Average	S.D.	*n*
Homozygous reference allele	4.40	2.91	16,418	3.36	1.28	3522
Homozygous alternative allele	4.06	0.80	743	2.94	1.02	3172
Heterozygous	5.17	0.39	106	5.25	3.26	14,639

*Note: n*, number of samples; S.D., standard deviation.

**FIGURE 3:**
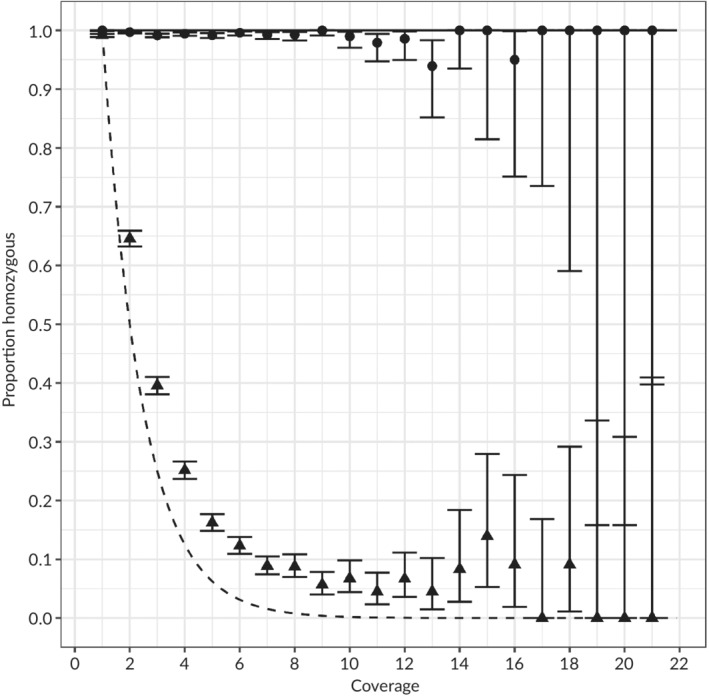
The experimentally observed proportions of homozygous female and male genotypes of SNPs associated with sex in Atlantic herring *Clupea harengus versus* read coverage (*x*) and the corresponding theoretically expected probabilities *P*(*x*|*hom*) = 1 and *P*(*x*|*het*) = 1/2^*x*− 1^. Error bars correspond to 95% confidence intervals from the binomial test. Observed: (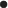) female, (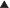) male; Expected: (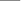) female, (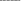) male

To investigate this further, the observed proportions of homozygous female and male genotypes *versus* coverage were compared with the corresponding theoretically expected probabilities *P*(*x*|*hom*) = 1 and *P*(*x*|*het*) = 1/2^*x*− 1^ (Chenuil, 2012) (Figure 3). The female proportions of homozygotes are all larger than 0.939 and 16 of them are larger than 0.990. The median is 0.996 (interquartile range = 0.008). Nine are exactly equal to 1 as expected for the females, while the binomial test rejects the null hypothesis for females in the remaining 12 of the 21 coverages (Supporting Information Table S1 and Figure 3). For the eight highest coverages of 14–21 only one is rejected. For males, the numerical discrepancies from the theoretical model are much larger and the binomial test rejects the null hypothesis for males in 17 of the 21 coverages, while we find an exact agreement for four of the five highest coverages of 17–21 (Supporting Information Table S3 and Figure 3). However, the overall trend for males is very different from the females and much more similar to the theoretical model, since the male homozygous proportion decreases towards zero for increasing coverage.

The experimental results for males indicate that perhaps not all the sex SNPs, but the majority must be heterozygous for males to develop. Nevertheless, these results support the suggestion of an XY sex determination system for *C. harengus*.

We see four possible explanations for the deviations from the theoretical expected proportions in Figure 3:

- The physiological sex has been wrongly registered. We think this explanation is unlikely because Figure 2 would then have indicated this with horizontal lines of the deviating zygosity.
- Random variations caused by a limited number of individuals tested. We cannot fully exclude this possibility, although we investigated 55 males.
- Some of the SNPs are not important for male sex determination and do not have to be present; they are mere passenger variations.
- A small proportion of the males have an alternative sex determination mechanism.

### 3.2 Search for possible sex determination genes

Because two regions were associated with sex, more than one gene could be involved in *C. harengus *sex determination. The region on chromosome 8 (21,063,400–22,268,779) contained 74 protein‐coding genes and the region on chromosome 21 (17,047,390–17,055,230) contained two protein‐coding genes. We investigated these genes for possible involvement in sex determination. None of these genes have previously been shown to be MSR genes in other organisms. However, to investigate further, possible orthologs for these genes were found and their reported functions investigated. None of the 76 genes were obvious candidates for being MSR genes, but 11 showed some potential linkage with sex determination or sex‐related functions. These are listed in Table 5, together with their possible link to sex determination. The orthologs of 8 of these 11 genes had noteworthy expression patterns or were X‐linked (Table 5). Among the remaining orthologs, the progesterone receptor membrane component 1 (*pgrmc1*) gene could potentially have a more convincing role in sex determination/differentiation processes as it is involved in oocyte maturation in *D. rerio* and plays a role in sexual maturation in male sea lamprey (*Petromyzon marinus*). Additionally, the *D. rerio* ortholog of the *C. harengus* trophoblast glycoprotein like (*tpbgl*) gene is wnt‐activated inhibitory factor 2 (*waif2*), which has been shown to be a modifier of Wnt signalling pathways (Table 5). Canonical wnt pathways are important for both mammalian and *D. rerio* sex determination (Harris *et al.*, 2018; Jordan *et al.*, 2001; Kossack *et al.*, 2019; Sreenivasan *et al.*, 2014).

**TABLE 5:** Atlantic herring *C. harengus* genes on the genomic regions associated with sex, together with their orthologs and possible link to sex determination or sex‐related functions

*C. harengus* gene	Orthologous gene	Orthologous species	Reason	Reference
*Smyd4*	*Smyd4*	*D. rerio*	Highest expression levels in testis	(Bastian *et al.*, 2008)
*Loc105890535*	*Macroh2a2*	*D. rerio*	Highest expression levels in testis	(Bastian *et al.*, 2008)
*Tpcn1*	*Tpcn1*	*D. rerio*	Highest expression levels in mature ovarian follicle	(Bastian *et al.*, 2008)
*Loc105890474*	*Nf2a*	*D. rerio*	Highest expression levels in mature ovarian follicle	(Bastian *et al.*, 2008)
*Loc105890446*	*NEXMIF*	*H. sapiens*	X‐linked	(Cason *et al.*, 2003)
*Sms*	*SMS*	*H. sapiens*	X‐linked	(Cantagrel *et al.*, 2004)
*Loc105890483*	*PRPS1L1*	*H. sapiens*	Specifically expressed in the testis	(Taira *et al.*, 1990)
	*Prps1b*	*D. rerio*	Expressed in 29 organs, with the highest expression level in mature ovarian follicles	(Bastian *et al.*, 2008)
*Iqcd*	*DRC1*	*H. sapiens*	Expressed in the testis and plays a role in fertilization	(Zhang *et al.*, 2019)
*Pgrmc1*	*Pgrmc1*	*D. rerio*	Plays a role in oocyte maturation	(Wu *et al.*, 2018)
		*Petromyzon marinus*	Plays a role in sexual maturation in male sea lamprey	(Bryan *et al.*, 2015)
*Tpbgl*	*Waif2*	*D. rerio*	*Waif2* has been shown to inhibit Wnt/β‐Catenin signalling and activate other wnt pathways	(Kagermeier‐Schenk *et al.*, 2011)
*Loc105911882*	*Bmpr1bb*	*D. rerio*	When mutated, fish have enlarged testes and accumulation of immature oocytes	(Neumann *et al.*, 2011)

To examine whether the sex SNPs could have functional consequences, their locations were investigated in more detail. Of these 529 SNPs, 151 were located in intergenic regions and 105 were located in promoter regions (Table 6). The SNPs in promoter regions could potentially affect the expression of genes. The remaining 273 SNPs were located in protein‐coding genes; however, the majority (167) were located in introns (Table 6). Among the 57 SNPs located in coding regions, 30 caused amino acid substitutions (Table 6). Among the 76 genes in the sex regions, six had sex SNPs that caused nonconservative amino acid substitutions in exons. These substitutions were in *tpcn1*, *iqcd*, *loc105890446*, claudin‐4‐like (*loc105890498*), *mettl27* and *bmpr1bb*. Table 7 lists the nonsynonymous SNPs and their corresponding amino acid substitutions. It is possible that these SNPs could have an effect on these genes, but to answer this question functional analyses need to be carried out.

**TABLE 6:** Location of the SNPs associated with sex in Atlantic herring *C. harengus*

Location of SNPs associated with sex	Number of SNPs
Intergenic regions	151
Promoter regions^a^	105
5′ untranslated regions	10
3′ untranslated regions	39
Splice sites	0
Introns	167
Coding regions	57
Synonymous SNPs	27
Nonsynonymous SNPs	30
Conservative amino acid substitutions	12
Nonconservative amino acid substitutions^b^	18

a 2000 bp upstream and 200 bp downstream of genes.

b Details of these nonconservative nonsynonymous substitutions are listed in Table 7.

**TABLE 7:** Nonconservative nonsynonymous substitutions in genes on the Atlantic herring *C. harengus* genome caused by SNPs significantly associated with sex

Gene	Chr	Pos	AA substitution	Changes to AA
*tpcn1*	8	21,072,591	Q‐ > H	Polar to positively charged
	8	21,077,772	S‐ > P	Polar to nonpolar
*iqcd*	8	21,086,634	Q‐ > K	Polar to positively charged
	8	21,088,137	S‐ > F	Polar to nonpolar
*loc105890446 (nexmifa)*	8	21,115,969	P‐ > S	Nonpolar to polar
	8	21,116,347	Q‐ > E	Nonpolar to negatively charged
	8	21,116,916	C‐ > Y	Nonpolar to polar
	8	21,117,352	N‐ > D	Polar to negatively charged
	8	21,117,963	S‐ > L	Nonpolar to polar
*loc105890498 (CLDN4)*	8	21,162,489	P‐ > T	Nonpolar to polar
*mettl27*	8	21,176,434	R‐ > S	Positively charged to polar
*loc105911882 (bmpr1bb)*	21	17,049,306	G‐ > S	Nonpolar to polar
	21	17,049,310	S‐ > L	Polar to nonpolar
	21	17,049,450	E‐ > K	Negatively charged to positively charged
	21	17,049,451	E‐ > A	Negatively charged to nonpolar
	21	17,049,466	Q‐ > L	Polar to nonpolar
	21	17,049,504	K‐ > E	Positively charged to negatively charged
	21	17,051,213	S‐ > A	polar to nonpolar

*Note:* Gene name abbreviations in parentheses are the Ensembl abbreviations and are only given if no abbreviations were available in GeneBank. AA, amino acid.

In addition, sex‐specific insertions and deletions (indels) in the two regions associated with sex, were investigated. After filtering there were 12 unique male indels and six unique female indels, all on chromosome 8 (Table 8). Seven of these indels (indels 1, 2, 5 and 13–16) were located in intragenic regions. Ten indels were located in the introns of the following genes: MAP/microtubule affinity‐regulating kinase 4 (*loc105890451*; indel 4), notchless protein homologue 1 (*nle1*; indels 6–10), uncharacterized LOC105890454 (*loc105890454*; indels 8–10; the *nle1* and *loc105890454* genes overlap but in opposite directions), melatonin receptor type 1B‐B (*loc105890457*; indels 11 and 12), connector enhancer of kinase suppressor of ras 2‐like (*loc105890461*; indel 17) and La ribonucleoprotein 1, translational regulator (*larp1*; indel 18) (Table 8). Furthermore, indel 3 was located in an exon of the gene protein NipSnap homologue 2‐like (*loc116221504*) and indel 8 was located in an exon of *loc105890454*. All indels except indel 4 caused frameshifts and therefore would most likely have a strong effect on the subsequent protein function. These indels are present in either all‐male or all‐female individuals (with data), so could play a role in sex development, however the genotypes of the indels vary within the sexes (Table 8). Most of the genotypes are homozygous for the alternative allele, but this could also be affected by the low sequencing coverage, as mentioned before.

**TABLE 8:** Sex‐specific deletions and insertions in genomic regions associated with sex in Atlantic herring *C. harengus*

Indel no.	Position	Male (M) or female (F) specific	Type	Indel size	Genotype counts (A/H)
1	CHR8:21,128,155	M	Deletion	1	34/6
2	CHR8:21,128,178	M	Deletion	1	38/2
3	CHR8:21,131,541	F	Insertion	2	30/4
4	CHR8:21,265,567	M	Insertion	3	34/5
5	CHR8:21,408,287	F	Insertion	1	32/2
6	CHR8:21,545,424	M	Deletion	1	34/7
7	CHR8:21,545,788	M	Deletion	1	41/1
8	CHR8:21,548,262	M	Deletion	1	34/6
9	CHR8:21,549,148	M	Insertion	1	39/6
10	CHR8:21,549,352	F	Insertion	1	34/2
11	CHR8:21,603,143	F	Deletion	1	29/6
12	CHR8:21,603,205	M	Insertion	1	37/7
13	CHR8:21,638,324	M	Deletion	2	38/1
14	CHR8:21,677,099	F	Insertion	1	32/5
15	CHR8:21,682,470	M	Insertion	2	33/6
16	CHR8:21,721,091	M	Deletion	1	37/6
17	CHR8:21,878,176	M	Insertion	2	38/10
18	CHR8:22,011,043	F	Insertion	2	29/7

*Note:* There were 55 males and 48 females but not all individuals had data for all variations because of the low sequencing coverage. Only insertions and deletions present in all individuals (with data) of the same sex were included. A, homozygous alternative allele; H, heterozygous.

None of the known sex determination or differentiation genes in fish were found on or close to the sex regions identified in this study. This could suggest that *C. harengus* has an unknown sex determination mechanism.

## 4 DISCUSSION

We identified two regions on two chromosomes on the *C. harengus* genome that were associated with sex. The data strongly indicated that females are homozygous, whereas the males are heterozygous for the SNPs in these sex‐associated regions. This is consistent with an XY sex determination system. There are 76 protein‐coding genes in these associated regions but no obvious MSR genes. However, some of these genes could potentially affect sex determination or development because they are associated with sex organs or sex functions in other species, as briefly referred to in the Results section (Table 5). Neither the investigation of the amino acid substitutions caused by SNPs nor that of indels pointed to a single sex determination gene in *C. harengus*.

### 4.1 Low sequencing coverage

The SNPs were identified by low coverage whole‐genome sequencing (on average 3 to 4× over the whole genome). This potentially resulted in some caveats regarding the genotypes. First, it is more likely to have missed genotypic data for some of the SNPs in some of the individuals, simply because the area has not been sequenced. Second, sequencing errors are more likely to be implemented as variations and could result in falsely called low‐frequency alleles. This is not a problem in the present situation because we are dealing with high‐frequency alleles. The third caveat is more serious: if, by chance, only one of the alleles from a heterozygous individual is sequenced, the genotype would always be called homozygous. With an average coverage of 3×, the probability of sequencing only one of the two alleles is on average 12.5% (and 12.5% for the other allele). Thus, statistically we would achieve a 75% detection rate in a group consisting of 100% heterozygotes (Chenuil, 2012). Our data were rather close to this theoretical expectation with 16.5% (3522/21,333) of male genotypes called homozygous reference alleles, another 14.9% (3172/21,333) homozygous alternative alleles and 68.6% (14,639/21,333) heterozygotes. Our results also showed that the male homozygous genotypes have on average lower coverage than the heterozygous genotypes, making it more probable that they are miscalled (Table 4). Furthermore, the observed male proportions of homozygotes *versus* coverage followed the same trend as the theoretically expected proportions if all genotypes were truly heterozygous (Figure 3). These results indicate that all the sex SNPs could potentially be heterozygotes in males.

One way to verify this would be to repeat the experiment with higher coverage. Meynert *et al*. (2014) demonstrated experimentally that 9–13× coverage was required to correctly call 95% of heterozygous genotypes. Chenuil (2012) showed that with a coverage of 5× (where all reads show the same allele) and a heterozygous rate of 0.5, the homozygous genotype would be correct 95% of the time. Therefore, a read depth of more than 5 would be appropriate to increase the sensitivity of correct genotypes to above 95% for both homo‐ and heterozygotes. The sex SNPs identified in this study could also be genotyped in genotyping experiments, rather than using sequencing.

In our study, the number of individuals sequenced partly makes up for the weakness caused by low coverage and shows that 99.4% of the female genotypes are homozygous, while at least 68.6% of the males are heterozygous. When genotyping an ideal male heterogametic sex‐determining system with sex‐linked SNP markers, we would expect females to be homozygous and males to be heterozygous at these markers. The very high proportion of homozygous females (99.4%) strongly supports this hypothesis, whereas the measured proportion of heterozygous males is limited by the much lower heterozygous sensitivity of the method at low coverage.

### 4.2 Sex regions on the *C. harengus* genome

The association between sex and chromosome 8:21,063,400–22,268,779 was stronger (*i.e*., lower *P* values) than for the region on chromosome 21, and it is also larger and contains more SNPs associated with sex (Table 2). As sex chromosomes evolve, they tend to become less stable and accumulate genes that are sex‐specific/beneficial, and eventually recombination between the homologous chromosomes stops and they become heteromorphic over time (Charlesworth *et al.*, 2005). However, not all species develop heteromorphic sex chromosomes (Wright *et al.*, 2016), for example the tiger pufferfish *Takifugu rubripes* (Temminck & Schlegel 1850) has only one SNP causing the phenotypic sex (Kamiya *et al.*, 2012). Our results showed that larger regions are associated with sex in *C. harengus*, but we cannot tell if these are early heteromorphic sex chromosomes in development or not.

It is interesting that two regions on different chromosomes are associated with sex in *C. harengus*. The genotypes of the sex SNPs on both regions show the same pattern (Figure 2). None of the female individuals investigated here have heterozygous genotypes at either region (with the exception of a few single SNPs). We would expect that the random segregation of chromosomes during meiosis would ensure that the different sex regions would sometimes end up in different gametes, thereby distributing among males and females in the offspring (assuming only two sexes in herring). This suggests that sex determination in herring could be complex, maybe polygenic (Moore & Roberts, 2013). Further studies are needed to investigate this possibility.

There are several possible explanations for the observation of two sex‐related regions located at different chromosomes:

- It might simply be a statistical coincidence. If so, the shorter region on chromosome 21 is most likely the one that is erroneously pointed out. However, as a rather conservative statistical threshold has been used, we think this is not a likely option.
- There might be an error in the chromosome assembly. A piece of chromosome 8 might have been assembled into chromosome 21, for example, because of similar repeated sequences at the two chromosomes. We have not been able to detect such repeated sequences. Still, assembly errors are rather common, even in high‐quality assemblies, so we will not exclude this possibility.
- If we assume that that it is biologically correct that there are two separate sex regions, and they segregate normally, we should have sometimes observed the heterozygous region of chromosome 8 and the homozygous region of chromosome 21 (and *vice versa*) in the same individual. This was never observed and would therefore only be explained by lethality of such mixes. This does not seem biologically plausible. Species with polygenic sex determination systems also tend to have a skewed sex ratio (Ser *et al.*, 2010), which is not true for *C. harengus*.
- It might be that chromosomes 8 and 21 are not segregating in the normal manner. There are a few examples of non‐Mendelian segregation, for example in the duck‐billed platypus (*Ornithorhynchus anatinus*) where five X chromosomes form a co‐segregating complex, and similarly with five Y chromosomes, resulting in a 1:1 sex ratio and only two possible genotypes (Rens *et al.*, 2004).

### 4.3 Potential genes involved in sex determination

As mentioned in the Results section, the genes on the sex regions are not known MSR genes or known to be part of the sex determination pathway, so a specific gene could not be identified as the most probable MSR gene. Therefore, the potential effect of sex SNPs was investigated further.

First, 105 sex SNPs were present in promoter regions and could alter the expression of these genes, thereby affecting the sex determination. However, expression studies would be needed to investigate this further. Second, the sex SNPs causing the nucleotide substitutions in the mRNA molecules could potentially cause the mRNA molecules to fold in different ways, making them less accessible by the ribosome and affecting the transcription of these mRNAs. Computational modelling of the folding needs to be done to test this possibility.

Third, the sex SNPs causing nonconservative amino acid substitutions could have an effect on the folding and function of the protein. For example, the *tpcn1* gene has two nonconservative amino acid substitutions caused by sex SNPs, at amino acid positions 245 and 537 (Table 7). No structural information is available for this gene/protein but aligning the *D. rerio tpcn1* with the *C. harengus tpcn1* shows extensive similarities. Amino acid positions 238 and 530 in the *D. rerio* protein correspond to positions 245 and 537 in the *C. harengus* protein and are within transmembrane domains. Nonconservative substitutions at these domains could disrupt the folding of the domains and affect the function of the protein. All the nonconservative amino acid substitutions caused by sex SNPs in the *C. harengus* gene/protein *loc105911882* (Table 7) are located in the protein kinase domain of the *D. rerio* protein. The substitution in the *C. harengus iqcd*, *loc105890446*, *loc105890498* and *mettl27* genes do not seem to be in any known domains of either the *C. harengus* or *D. rerio* proteins.

There could potentially still be protein coding genes that have not yet been predicted. This is partly because of potentially suboptimal prediction algorithms and partly because they could be within nonsequenced regions. Moreover, it is likely that many nonidentified noncoding genes exist, such as lncRNAs or miRNAs. In addition to genes, there are many regulatory elements that are not necessarily close to the genes they regulate. *C. harengus* is not a model organism, and thus there have been limited studies with this species, but the ENCODE project has inferred many functions for noncoding parts of the *H. sapiens* genome (ENCODE Project Consortium, 2012). It is highly likely that similar noncoding elements exist in the *C. harengus* genome, and some of the SNPs found in this study to be associated with sex could affect a noncoding element that has not been identified yet.

In addition, there might be sex‐specific sequences present in *C. harengus*. For example, if the reference genome assembly was from a female individual and *C. harengus* has a male‐specific sequence that controls sex determination, then this study would not be able to identify this. It is likely that this is the case, and that the sex‐specific sequence is at or close to the sex regions identified here, and the heterozygous male sex SNPs are linked with this sex‐specific sequence. Targeted sequencing of these regions could reveal if this is true.

### 4.4 Evolution of sex determination within the Clupeiformes order

Teleost fishes have highly diverse sex determination systems (Bachtrog *et al.*, 2014). The XY sex determination system for *C. harengus*, suggested in this study, fits well with the other Clupeiformes mentioned in the Introduction.

A study by Pennell *et al*. (2018) indicated that in fish, transitions from gonochorism to hermaphroditism occur at higher rates than the reverse, and transitions from female to male heterogamety occur at higher rates than the reverse. They also found similar transition rates between homomorphic and heteromorphic sex chromosomes in both fish and amphibians. This could suggest that the common ancestor for Clupeidae and Engraulidae had a Z0 or ZW sex determination system, which is still present in *Coilia nasus* (Xu *et al.*, 2014). The common ancestor for Clupeidae then lost the Z chromosome and adapted to a XY system, which has been found in *Brevoortia* and now also in *Clupea*. These sex chromosomes are early in their evolution and still homomorphic, as seen in *Brevoortia* spp. and *C. harengus*. A single known exception is *B. aurea*, a species that has heteromorphic sex chromosomes with two X and one Y chromosome. As *Tenulosa* split from *Brevoortia* and *Clupea*, they evolved to be hermaphrodites. Of course, this series of events is a speculative hypothesis at present.

### 4.5 Conclusion and future work

We identified regions on the *C. harengus* genome that were associated with sex. The genotypes of the SNPs associated with sex indicated an XY sex determination system for *C. harengus*, which is consistent with other Clupeiformes species. Nonetheless, we could not identify the exact genes for sex determination. None of the known sex determination genes in fish were found on or close to the sex regions, indicating that *C. harengus* could have a previously unregistered, unknown sex determination mechanism. New experiments where these sex regions are sequenced at a higher coverage for both males and females should be conducted to reproduce and more effectively delineate the sex determination regions. This would also better characterize the potential existence of homozygous SNPs in a small proportion of the males in these regions and could identify possible sex‐specific sequences.

## AUTHOR CONTRIBUTIONS

S.í.K. designed the study, conducted the laboratory work, analysed and interpreted the data, and wrote the manuscript. S.O.M. contributed to the design of the study and writing of the manuscript, and supervised the laboratory work and analysis and interpretation of data. E.í.H. and J.A.J. contributed to the acquisition and interpretation of the data. H.G. contributed to the statistical analysis and interpretation of the data, acquired funding, and contributed to the writing of the manuscript. P.F. contributed to the design of the study, writing of the manuscript and analysis and interpretation of the data. H.A.D. designed the study, acquired funding, contributed to the writing of the manuscript and supervised the laboratory work and analysis and interpretation of data. All authors contributed to revising the manuscript and approved the final version.

## Supporting information


**SUPPORTING INFORMATION TABLE S1** List of sex determination genes searched for in the Atlantic herring genome
**SUPPORTING INFORMATION TABLE S2** List of SNPs associated with sex in Atlantic herring
**SUPPORTING INFORMATION TABLE S3** Test results from the comparison of the observed proportions of homozygous female and male genotypes *versus* coverage with the corresponding theoretically expected probabilities

## References

[jfb14349-bib-0001] Allsop, D. J. , & West, S. A. (2003). Constant relative age and size at sex change for sequentially hermaphroditic fish. Journal of Evolutionary Biology, 16, 921–929. 14635907 10.1046/j.1420-9101.2003.00590.x

[jfb14349-bib-0002] Andrews, S. (2010). FastQC: A quality control tool for high throughput sequence data. Retrieved from: http://www.bioinformatics.babraham.ac.uk/projects/fastqc

[jfb14349-bib-0003] Ashman, T.‐L. , Bachtrog, D. , Blackmon, H. , Goldberg, E. E. , Hahn, M. W. , Kirkpatrick, M. , … Ming, R. (2014). Tree of sex: A database of sexual systems. Scientific Data, 1, 140015. 25977773 10.1038/sdata.2014.15 PMC4322564

[jfb14349-bib-0004] Bachtrog, D. , Mank, J. E. , Peichel, C. L. , Kirkpatrick, M. , Otto, S. P. , Ashman, T.‐L. , … Ming, R. (2014). Sex determination: Why so many ways of doing it? PLoS Biology, 12, e1001899. 24983465 10.1371/journal.pbio.1001899 PMC4077654

[jfb14349-bib-0005] Baroiller, J.‐F. , D'Cotta, H. , Bezault, E. , Wessels, S. , & Hoerstgen‐Schwark, G. (2009). Tilapia sex determination: Where temperature and genetics meet. Comparative Biochemistry and Physiology Part A: Molecular & Integrative Physiology, 153, 30–38. 10.1016/j.cbpa.2008.11.018 19101647

[jfb14349-bib-0006] Bastian, F. , Parmentier, G. , Roux, J. , Moretti, S. , Laudet, V. , & Robinson‐Rechavi, M. (2008). Bgee: Integrating and comparing heterogeneous transcriptome data among species In International Workshop on Data Integration in the Life Sciences (pp. 124–131). Berlin, Heidelberg: Springer.

[jfb14349-bib-0007] Bolger, A. M. , Lohse, M. , & Usadel, B. (2014). Trimmomatic: A flexible trimmer for Illumina sequence data. Bioinformatics, 30, 2114–2120. 24695404 10.1093/bioinformatics/btu170 PMC4103590

[jfb14349-bib-0008] Brum, M. (1992). Multiple sex chromosomes in South Atlantic fish, *Brevoortia aurea*, Clupeidae. Brazilian Journal of Genetics, 15, 547–553.

[jfb14349-bib-0009] Bryan, M. B. , Chung‐Davidson, Y.‐W. , Ren, J. , Bowman, S. , Scott, A. P. , Huertas, M. , … Li, W. (2015). Evidence that progestins play an important role in spermiation and pheromone production in male sea lamprey (*Petromyzon marinus*). General and Comparative Endocrinology, 212, 17–27. 25623147 10.1016/j.ygcen.2015.01.008

[jfb14349-bib-0010] Brykov, V. A. (2014). Mechanisms of sex determination in fish: Evolutionary and practical aspects. Russian Journal of Marine Biology, 40, 407–417.

[jfb14349-bib-0011] Bull, J. J. (1983). Evolution of sex determining mechanisms. Menlo Park, CA: The Benjamin/Cummings Publishing Company, Inc.

[jfb14349-bib-0012] Buston, P. (2003). Social hierarchies: Size and growth modification in clownfish. Nature, 424, 145–146. 12853944 10.1038/424145a

[jfb14349-bib-0013] Camacho, C. , Coulouris, G. , Avagyan, V. , Ma, N. , Papadopoulos, J. , Bealer, K. , & Madden, T. L. (2009). BLAST+: Architecture and applications. BMC Bioinformatics, 10, 421. 20003500 10.1186/1471-2105-10-421 PMC2803857

[jfb14349-bib-0014] Cantagrel, V. , Lossi, A. , Boulanger, S. , Depetris, D. , Mattei, M. , Gecz, J. , … Villard, L. (2004). Disruption of a new X linked gene highly expressed in brain in a family with two mentally retarded males. Journal of Medical Genetics, 41, 736–742. 15466006 10.1136/jmg.2004.021626 PMC1735597

[jfb14349-bib-0015] Cason, A. L. , Ikeguchi, Y. , Skinner, C. , Wood, T. C. , Holden, K. R. , Lubs, H. A. , … Pegg, A. E. (2003). X‐linked spermine synthase gene (*SMS*) defect: The first polyamine deficiency syndrome. European Journal of Human Genetics, 11, 937–944. 14508504 10.1038/sj.ejhg.5201072

[jfb14349-bib-0016] Charlesworth, D. , Charlesworth, B. , & Marais, G. (2005). Steps in the evolution of heteromorphic sex chromosomes. Heredity, 95, 118–128. 15931241 10.1038/sj.hdy.6800697

[jfb14349-bib-0017] Chen, S. , Zhang, G. , Shao, C. , Huang, Q. , Liu, G. , Zhang, P. , … Volff, J.‐N. (2014). Whole‐genome sequence of a flatfish provides insights into ZW sex chromosome evolution and adaptation to a benthic lifestyle. Nature Genetics, 46, 253–260. 24487278 10.1038/ng.2890

[jfb14349-bib-0018] Chenuil, A. (2012). How to infer reliable diploid genotypes from NGS or traditional sequence data: from basic probability to experimental optimization. Journal of Evolutionary Biology, 25, 949–960. 22420488 10.1111/j.1420-9101.2012.02488.x

[jfb14349-bib-0019] Clinton, M. (1998). Sex determination and gonadal development: A bird's eye view. Journal of Experimental Zoology, 281, 457–465. 9662832 10.1002/(sici)1097-010x(19980801)281:5<457::aid-jez10>3.0.co;2-6

[jfb14349-bib-0020] Devlin, R. H. , & Nagahama, Y. (2002). Sex determination and sex differentiation in fish: An overview of genetic, physiological, and environmental influences. Aquaculture, 208, 191–364.

[jfb14349-bib-0021] Doucette, A. J., Jr. , & Fitzsimons, J. M. (1988). Karyology of elopiform and clupeiform fishes. Copeia, 1988, 124–130.

[jfb14349-bib-0022] ENCODE Project Consortium . (2012). An integrated encyclopedia of DNA elements in the human genome. Nature, 489, 57–74. 22955616 10.1038/nature11247 PMC3439153

[jfb14349-bib-0023] Fadista, J. , Manning, A. K. , Florez, J. C. , & Groop, L. (2016). The (in) famous GWAS P‐value threshold revisited and updated for low‐frequency variants. European Journal of Human Genetics, 24, 1202–1205. 26733288 10.1038/ejhg.2015.269 PMC4970684

[jfb14349-bib-0024] Fricke, H. W. (1979). Mating system, resource defence and sex change in the anemonefish *Amphiprion akallopisos* . Zeitschrift für Tierpsychologie, 50, 313–326.

[jfb14349-bib-0025] Furuta, T. , Ashikari, M. , Jena, K. K. , Doi, K. , & Reuscher, S. (2017). Adapting genotyping‐by‐sequencing for rice F2 populations. G3: Genes, Genomes, Genetics, 7, 881–893. 28082325 10.1534/g3.116.038190 PMC5345719

[jfb14349-bib-0026] Garrison, E. & Marth, G. (2012). Haplotype‐based variant detection from short‐read sequencing. *arXiv preprint arXiv:1207.3907*.

[jfb14349-bib-0027] Harris, A. , Siggers, P. , Corrochano, S. , Warr, N. , Sagar, D. , Grimes, D. T. , … Koo, B.‐K. (2018). ZNRF3 functions in mammalian sex determination by inhibiting canonical WNT signaling. Proceedings of the National Academy of Sciences of the United States of America, 115, 5474–5479. 29735715 10.1073/pnas.1801223115 PMC6003506

[jfb14349-bib-0028] Hattori, R. S. , Murai, Y. , Oura, M. , Masuda, S. , Majhi, S. K. , Sakamoto, T. , … Strüssmann, C. A. (2012). A Y‐linked anti‐Müllerian hormone duplication takes over a critical role in sex determination. Proceedings of the National Academy of Sciences of the United States of America, 109, 2955–2959. 22323585 10.1073/pnas.1018392109 PMC3286941

[jfb14349-bib-0029] Jordan, B. K. , Mohammed, M. , Ching, S. T. , Délot, E. , Chen, X.‐N. , Dewing, P. , … Vilain, E. (2001). Up‐regulation of WNT‐4 signaling and dosage‐sensitive sex reversal in humans. The American Journal of Human Genetics, 68, 1102–1109. 11283799 10.1086/320125 PMC1226091

[jfb14349-bib-0030] Kagermeier‐Schenk, B. , Wehner, D. , Özhan‐Kizil, G. , Yamamoto, H. , Li, J. , Kirchner, K. , … Schambony, A. (2011). Waif1/5T4 inhibits Wnt/β‐catenin signaling and activates noncanonical Wnt pathways by modifying LRP6 subcellular localization. Developmental Cell, 21, 1129–1143. 22100263 10.1016/j.devcel.2011.10.015

[jfb14349-bib-0031] Kamiya, T. , Kai, W. , Tasumi, S. , Oka, A. , Matsunaga, T. , Mizuno, N. , … Hosoya, S. (2012). A trans‐species missense SNP in *Amhr2* is associated with sex determination in the tiger pufferfish, *Takifugu rubripes* (fugu). PLoS Genetics, 8, e1002798. 22807687 10.1371/journal.pgen.1002798 PMC3395601

[jfb14349-bib-0032] Kashimada, K. , & Koopman, P. (2010). Sry: the master switch in mammalian sex determination. Development, 137, 3921–3930. 21062860 10.1242/dev.048983

[jfb14349-bib-0033] Kossack, M. E. , High, S. K. , Hopton, R. E. , Yan, Y. L. , Postlethwait, J. H. , & Draper, B. W. (2019). Female sex development and reproductive duct formation depend on Wnt4a in zebrafish. Genetics, 211, 219–233. 30446521 10.1534/genetics.118.301620 PMC6325708

[jfb14349-bib-0034] Kriventseva, E. V. , Kuznetsov, D. , Tegenfeldt, F. , Manni, M. , Dias, R. , Simão, F. A. , & Zdobnov, E. M. (2019). OrthoDB v10: Sampling the diversity of animal, plant, fungal, protist, bacterial and viral genomes for evolutionary and functional annotations of orthologs. Nucleic Acids Research, 47, D807–D811. 30395283 10.1093/nar/gky1053 PMC6323947

[jfb14349-bib-0035] Lagomarsino, I. V. , & Conover, D. O. (1993). Variation in environmental and genotypic sex‐determining mechanisms across a latitudinal gradient in the fish, *Menidia menidia* . Evolution, 47, 487–494. 28568738 10.1111/j.1558-5646.1993.tb02108.x

[jfb14349-bib-0036] Li, H. (2013). Aligning sequence reads, clone sequences and assembly contigs with BWA‐MEM. *arXiv preprint arXiv:1303.3997*.

[jfb14349-bib-0037] Li, H. , Handsaker, B. , Wysoker, A. , Fennell, T. , Ruan, J. , Homer, N. , … Durbin, R. (2009). The sequence alignment/map format and SAMtools. Bioinformatics, 25, 2078–2079. 19505943 10.1093/bioinformatics/btp352 PMC2723002

[jfb14349-bib-0038] Liu, H. , Pang, M. , Yu, X. , Zhou, Y. , Tong, J. , & Fu, B. (2018). Sex‐specific markers developed by next‐generation sequencing confirmed an XX/XY sex determination system in bighead carp (*Hypophthalmichthys nobilis*) and silver carp (*Hypophthalmichthys molitrix*). DNA Research, 25, 257–264. 10.1093/dnares/dsx054 PMC6014435 29315393

[jfb14349-bib-0039] Meynert, A. M. , Ansari, M. , FitzPatrick, D. R. , & Taylor, M. S. (2014). Variant detection sensitivity and biases in whole genome and exome sequencing. BMC Bioinformatics, 15, 247. 25038816 10.1186/1471-2105-15-247 PMC4122774

[jfb14349-bib-0040] Moore, E. C. , & Roberts, R. B. (2013). Polygenic sex determination. Current Biology, 23, R510–R512. 23787041 10.1016/j.cub.2013.04.004

[jfb14349-bib-0041] Neumann, J. C. , Chandler, G. L. , Damoulis, V. A. , Fustino, N. J. , Lillard, K. , Looijenga, L. , … Amatruda, J. F. (2011). Mutation in the type IB bone morphogenetic protein receptor Alk6b impairs germ‐cell differentiation and causes germ‐cell tumors in zebrafish. Proceedings of the National Academy of Sciences of the United States of America, 108, 13153–13158. 21775673 10.1073/pnas.1102311108 PMC3156187

[jfb14349-bib-0042] Obenchain, V. , Lawrence, M. , Carey, V. , Gogarten, S. , Shannon, P. , & Morgan, M. (2014). VariantAnnotation: A Bioconductor package for exploration and annotation of genetic variants. Bioinformatics, 30, 2076–2078. 24681907 10.1093/bioinformatics/btu168 PMC4080743

[jfb14349-bib-0043] Palaiokostas, C. , Bekaert, M. , Taggart, J. B. , Gharbi, K. , McAndrew, B. J. , Chatain, B. , … Vandeputte, M. (2015). A new SNP‐based vision of the genetics of sex determination in European sea bass (*Dicentrarchus labrax*). Genetics Selection Evolution, 47, 68. 10.1186/s12711-015-0148-y PMC4558911 26337592

[jfb14349-bib-0044] Pennell, M. W. , Mank, J. E. , & Peichel, C. L. (2018). Transitions in sex determination and sex chromosomes across vertebrate species. Molecular Ecology, 27, 3950–3963. 29451715 10.1111/mec.14540 PMC6095824

[jfb14349-bib-0045] Pieau, C. (1996). Temperature variation and sex determination in reptiles. BioEssays, 18, 19–26.

[jfb14349-bib-0046] Purcell, C. M. , Seetharam, A. S. , Snodgrass, O. , Ortega‐García, S. , Hyde, J. R. , & Severin, A. J. (2018). Insights into teleost sex determination from the *Seriola dorsalis* genome assembly. BMC Genomics, 19, 31. 29310588 10.1186/s12864-017-4403-1 PMC5759298

[jfb14349-bib-0047] Purcell, S. , Neale, B. , Todd‐Brown, K. , Thomas, L. , Ferreira, M. A. , Bender, D. , … Daly, M. J. (2007). PLINK: A tool set for whole‐genome association and population‐based linkage analyses. The American Journal of Human Genetics, 81, 559–575. 17701901 10.1086/519795 PMC1950838

[jfb14349-bib-0048] Reed, E. , Nunez, S. , Kulp, D. , Qian, J. , Reilly, M. P. , & Foulkes, A. S. (2015). A guide to genome‐wide association analysis and post‐analytic interrogation. Statistics in Medicine, 34, 3769–3792. 26343929 10.1002/sim.6605 PMC5019244

[jfb14349-bib-0049] Rens, W. , Grützner, F. , O'Brien, P. C. , Fairclough, H. , Graves, J. A. , & Ferguson‐Smith, M. A. (2004). Resolution and evolution of the duck‐billed platypus karyotype with an X1Y1X2Y2X3Y3X4Y4X5Y5 male sex chromosome constitution. Proceedings of the National Academy of Sciences of the United States of America, 101, 16257–16261. 15534209 10.1073/pnas.0405702101 PMC528943

[jfb14349-bib-0050] Roberts, N. B. , Juntti, S. A. , Coyle, K. P. , Dumont, B. L. , Stanley, M. K. , Ryan, A. Q. , … Roberts, R. B. (2016). Polygenic sex determination in the cichlid fish *Astatotilapia burtoni* . BMC Genomics, 17, 835. 27784286 10.1186/s12864-016-3177-1 PMC5080751

[jfb14349-bib-0051] Ser, J. R. , Roberts, R. B. , & Kocher, T. D. (2010). Multiple interacting loci control sex determination in Lake Malawi cichlid fish. Evolution: International Journal of Organic Evolution, 64, 486–501. 19863587 10.1111/j.1558-5646.2009.00871.x PMC3176681

[jfb14349-bib-0052] Shen, Z.‐G. , & Wang, H.‐P. (2014). Molecular players involved in temperature‐dependent sex determination and sex differentiation in Teleost fish. Genetics Selection Evolution, 46, 26. 10.1186/1297-9686-46-26 PMC4108122 24735220

[jfb14349-bib-0053] Sreenivasan, R. , Jiang, J. , Wang, X. , Bártfai, R. , Kwan, H. Y. , Christoffels, A. , & Orbán, L. (2014). Gonad differentiation in zebrafish is regulated by the canonical Wnt signaling pathway. Biology of Reproduction, 90(45), 41–10. 24174574 10.1095/biolreprod.113.110874

[jfb14349-bib-0054] Sun, C. , Niu, Y. , Ye, X. , Dong, J. , Hu, W. , Zeng, Q. , … Lu, M. (2017). Construction of a high‐density linkage map and mapping of sex determination and growth‐related loci in the mandarin fish (*Siniperca chuatsi*). BMC Genomics, 18, 446. 28587594 10.1186/s12864-017-3830-3 PMC5461734

[jfb14349-bib-0055] Taira, M. , Iizasa, T. , Shimada, H. , Kudoh, J. , Shimizu, N. , & Tatibana, M. (1990). A human testis‐specific mRNA for phosphoribosylpyrophosphate synthetase that initiates from a non‐AUG codon. Journal of Biological Chemistry, 265, 16491–16497. 2168892

[jfb14349-bib-0056] Turner, (2018). qqman: an R package for visualizing GWAS results using Q‐Q and manhattan plots. *Journal of Open Source Software*, *3*(25), 731.

[jfb14349-bib-0057] Wilson, C. A. , High, S. K. , McCluskey, B. M. , Amores, A. , Yan, Y. L. , Titus, T. A. , … Schartl, M. (2014). Wild sex in zebrafish: Loss of the natural sex determinant in domesticated strains. Genetics, 198, 1291–1308. 25233988 10.1534/genetics.114.169284 PMC4224167

[jfb14349-bib-0058] Wright, A. E. , Dean, R. , Zimmer, F. , & Mank, J. E. (2016). How to make a sex chromosome. Nature Communications, 7, 12087. 10.1038/ncomms12087 PMC4932193 27373494

[jfb14349-bib-0059] Wu, X.‐J. , Thomas, P. , & Zhu, Y. (2018). *Pgrmc1* knockout impairs oocyte maturation in zebrafish. Frontiers in Endocrinology, 9, 560. 30319543 10.3389/fendo.2018.00560 PMC6165893

[jfb14349-bib-0060] Xu, S. , Li, Y. , Fu, G. , Wu, H. W. , Wang, Q. , Liu, Q. G. , & Qu, X. C. (2014). Chromosome karyotype analysis of *Coilia nasus* . Guangdong Agricultural Sciences, 7, 155–157.

[jfb14349-bib-0061] Yano, A. , Guyomard, R. , Nicol, B. , Jouanno, E. , Quillet, E. , Klopp, C. , … Guiguen, Y. (2012). An immune‐related gene evolved into the master sex‐determining gene in rainbow trout, *Oncorhynchus mykiss* . Current Biology, 22, 1423–1428. 22727696 10.1016/j.cub.2012.05.045

[jfb14349-bib-0062] Yoshimoto, S. , & Ito, M. (2011). A ZZ/ZW‐type sex determination in *Xenopus laevis* . The FEBS Journal, 278, 1020–1026. 21281450 10.1111/j.1742-4658.2011.08031.x

[jfb14349-bib-0063] Zhang, P. , Jiang, W. , Luo, N. , Zhu, W. , & Fan, L. (2019). Corrigendum to: IQ motif containing D (IQCD), a new acrosomal protein involved in the acrosome reaction and fertilisation. Reproduction, Fertility and Development, 31, 1033–1033. 10.1071/RD18416_CO 31039996

