# Supplementary material for: Identification of male heterogametic sex‐determining regions on the Atlantic herring Clupea harengus genome
Source: J Fish Biol. 2020 May 22;97(1):190–201. doi: 10.1111/jfb.14349 (PMC7115899; doi:10.1111/jfb.14349)
Supplement: Supplementary file 1 — SUPPORTING INFORMATION TABLE S1 List of sex determination genes searched for in the Atlantic herring genome SUPPORTING INFORMATION TABLE S2 List of SNPs associated with sex in Atlantic herring SUPPORTING INFORMATION TABLE S3 Test results from the comparison of the observed proportions of homozygous female and male genotypes versus coverage with the corresponding theoretically expected probabilities [file JFB-97-190-s001.docx]

**Supplementary material**

**Supplementary Table S1. List of sex determination genes that were searched for on the Atlantic herring (*Clupea harengus*) genome.** The sequence used in the search and the assembly version which the sequence was taken from are listed in columns three and four, respectively.

| **Gene** | **Species** | **Genomic sequence** | **Assembly version** |
| --- | --- | --- | --- |
| *Cyp19a1a* | *Danio rerio* | NC_007129.7:c39636348-39620534 | GRCz11 |
| *Cyp19a1b* | *Oryzias latipes* | NC_019864.2:c15396898-15393093 | ASM223467v1 |
| *Sox3* | *Danio rerio* | NC_007125.7:c32744464-32742701 | GRCz11 |
| *Sox5* | *Danio rerio* | NC_007115.7:c17244201-16981414 | GRCz11 |
| *Sox9* | *Danio rerio* | NC_007123.7:c1951233-1947593 | GRCz11 |
| *DmY* | *Oryzias latipes* | NC_019867.2:c1215534-1177806 | ASM223467v1 |
| *Wnt4* | *Danio rerio* | NC_007122.7:c39202915-39167846 | GRCz11 |
| *Irf9* | *Danio rerio* | NC_007123.7:13282709-13306886 | GRCz11 |
| *B-catenin* | *Cynoglossus semilaevis* | NC_024319.1:c6052535-6041341 | Cse_v1.0 |
| *Fgf9* | *Danio rerio* | NC_007120.7:c19611480-19604372 | GRCz11 |
| *Foxl2* | *Kryptolebias marmoratus* | NW_016094302.1:c134814-132896 | ASM164957v1 |
| *Gsdf* | *Danio rerio* | NC_007132.7:196490-205221 | GRCz11 |
| *Amhy* | *Oreochromis niloticus* | NC_031986.2:c34502800-34498945 | O_niloticus_UMD_NMBU |
| *Dmrt* | *Danio rerio* | NC_007116.7:44944778-44991344 | GRCz11 |
| *Sdy* | *Oncorhynchus mykiss* | NW_018580030.1:c40458-32753 | Omyk_1.0 |
| *Amhr2* | *Takifugu rubripes* | NC_042303.1:12703026-12709063 | fTakRub1.2 |
| *Amh* | *Oreochromis niloticus* | NC_031986.2:c34502800-34498945 | O_niloticus_UMD_NMBU |

**Supplementary Table S2. List of SNPs significantly associated with sex in Atlantic herring (*Clupea harengus*) found in a GWAS.**

| **CHR** | **POS** | **REF** | **ALT** |
| --- | --- | --- | --- |
| LR535864.1 | 21063400 | C | G |
| LR535864.1 | 21063798 | G | C |
| LR535864.1 | 21063813 | A | G |
| LR535864.1 | 21063832 | C | T |
| LR535864.1 | 21063866 | A | T |
| LR535864.1 | 21063895 | C | T |
| LR535864.1 | 21063934 | G | A |
| LR535864.1 | 21063944 | G | A |
| LR535864.1 | 21064085 | T | A |
| LR535864.1 | 21064117 | T | C |
| LR535864.1 | 21064236 | G | A |
| LR535864.1 | 21064305 | A | G |
| LR535864.1 | 21064320 | G | A |
| LR535864.1 | 21064332 | A | T |
| LR535864.1 | 21065483 | G | A |
| LR535864.1 | 21065489 | G | T |
| LR535864.1 | 21066774 | G | A |
| LR535864.1 | 21066796 | T | A |
| LR535864.1 | 21066817 | C | T |
| LR535864.1 | 21066851 | G | C |
| LR535864.1 | 21066881 | C | T |
| LR535864.1 | 21066907 | A | C |
| LR535864.1 | 21066912 | G | T |
| LR535864.1 | 21066919 | C | T |
| LR535864.1 | 21066950 | A | C |
| LR535864.1 | 21066951 | A | G |
| LR535864.1 | 21066973 | T | A |
| LR535864.1 | 21067003 | G | A |
| LR535864.1 | 21067021 | T | A |
| LR535864.1 | 21067026 | T | C |
| LR535864.1 | 21067056 | A | T |
| LR535864.1 | 21067135 | T | C |
| LR535864.1 | 21067140 | C | T |
| LR535864.1 | 21067191 | T | C |
| LR535864.1 | 21067263 | A | T |
| LR535864.1 | 21067268 | G | T |
| LR535864.1 | 21067270 | A | C |
| LR535864.1 | 21067281 | A | T |
| LR535864.1 | 21067299 | A | T |
| LR535864.1 | 21067304 | A | G |
| LR535864.1 | 21067321 | A | T |
| LR535864.1 | 21067334 | C | T |
| LR535864.1 | 21067404 | C | G |
| LR535864.1 | 21067418 | C | T |
| LR535864.1 | 21067483 | C | G |
| LR535864.1 | 21067487 | G | T |
| LR535864.1 | 21067489 | G | A |
| LR535864.1 | 21067500 | C | G |
| LR535864.1 | 21067533 | T | C |
| LR535864.1 | 21067536 | G | A |
| LR535864.1 | 21068369 | G | A |
| LR535864.1 | 21068381 | T | C |
| LR535864.1 | 21068395 | G | T |
| LR535864.1 | 21068427 | G | T |
| LR535864.1 | 21068442 | T | A |
| LR535864.1 | 21068575 | A | T |
| LR535864.1 | 21068593 | C | A |
| LR535864.1 | 21068613 | T | C |
| LR535864.1 | 21068616 | A | G |
| LR535864.1 | 21068617 | A | T |
| LR535864.1 | 21068628 | G | A |
| LR535864.1 | 21068629 | G | C |
| LR535864.1 | 21068633 | A | T |
| LR535864.1 | 21068659 | C | T |
| LR535864.1 | 21068685 | A | G |
| LR535864.1 | 21068885 | A | G |
| LR535864.1 | 21069014 | G | T |
| LR535864.1 | 21069105 | T | C |
| LR535864.1 | 21069136 | C | G |
| LR535864.1 | 21069169 | T | C |
| LR535864.1 | 21069217 | T | G |
| LR535864.1 | 21069219 | A | T |
| LR535864.1 | 21069961 | C | T |
| LR535864.1 | 21069968 | T | C |
| LR535864.1 | 21070016 | G | C |
| LR535864.1 | 21070034 | C | A |
| LR535864.1 | 21070036 | G | T |
| LR535864.1 | 21070089 | A | G |
| LR535864.1 | 21070111 | G | A |
| LR535864.1 | 21070114 | A | T |
| LR535864.1 | 21070143 | T | C |
| LR535864.1 | 21070222 | T | C |
| LR535864.1 | 21070258 | T | G |
| LR535864.1 | 21070297 | A | C |
| LR535864.1 | 21070344 | C | A |
| LR535864.1 | 21071113 | A | C |
| LR535864.1 | 21071135 | C | A |
| LR535864.1 | 21071229 | A | T |
| LR535864.1 | 21071402 | C | T |
| LR535864.1 | 21071589 | G | A |
| LR535864.1 | 21071610 | C | T |
| LR535864.1 | 21071620 | G | C |
| LR535864.1 | 21071722 | C | A |
| LR535864.1 | 21071812 | G | T |
| LR535864.1 | 21071862 | T | C |
| LR535864.1 | 21071920 | T | A |
| LR535864.1 | 21072464 | A | C |
| LR535864.1 | 21072591 | T | G |
| LR535864.1 | 21072654 | T | G |
| LR535864.1 | 21072901 | A | G |
| LR535864.1 | 21073228 | G | A |
| LR535864.1 | 21073254 | C | T |
| LR535864.1 | 21073274 | T | C |
| LR535864.1 | 21073293 | T | A |
| LR535864.1 | 21073296 | G | C |
| LR535864.1 | 21073319 | A | G |
| LR535864.1 | 21073529 | C | G |
| LR535864.1 | 21073543 | G | T |
| LR535864.1 | 21074025 | G | T |
| LR535864.1 | 21074149 | A | G |
| LR535864.1 | 21074185 | A | C |
| LR535864.1 | 21074411 | A | G |
| LR535864.1 | 21074622 | G | T |
| LR535864.1 | 21074706 | T | A |
| LR535864.1 | 21074727 | C | A |
| LR535864.1 | 21074753 | G | T |
| LR535864.1 | 21074780 | T | G |
| LR535864.1 | 21075016 | T | A |
| LR535864.1 | 21075048 | A | T |
| LR535864.1 | 21075160 | C | A |
| LR535864.1 | 21075177 | G | T |
| LR535864.1 | 21075285 | A | G |
| LR535864.1 | 21076352 | G | T |
| LR535864.1 | 21076377 | A | T |
| LR535864.1 | 21076572 | G | A |
| LR535864.1 | 21076762 | A | C |
| LR535864.1 | 21076774 | G | A |
| LR535864.1 | 21077243 | A | G |
| LR535864.1 | 21077532 | A | T |
| LR535864.1 | 21077533 | C | T |
| LR535864.1 | 21077641 | G | A |
| LR535864.1 | 21077727 | G | T |
| LR535864.1 | 21077772 | A | G |
| LR535864.1 | 21078421 | C | T |
| LR535864.1 | 21079445 | C | T |
| LR535864.1 | 21079675 | G | A |
| LR535864.1 | 21079685 | A | G |
| LR535864.1 | 21079991 | G | C |
| LR535864.1 | 21080084 | T | A |
| LR535864.1 | 21080378 | A | G |
| LR535864.1 | 21080447 | T | C |
| LR535864.1 | 21080670 | G | A |
| LR535864.1 | 21080717 | G | A |
| LR535864.1 | 21080748 | C | T |
| LR535864.1 | 21081320 | C | G |
| LR535864.1 | 21081332 | C | T |
| LR535864.1 | 21081337 | G | A |
| LR535864.1 | 21081345 | A | C |
| LR535864.1 | 21081347 | T | A |
| LR535864.1 | 21081605 | G | A |
| LR535864.1 | 21081940 | A | C |
| LR535864.1 | 21081985 | T | C |
| LR535864.1 | 21082151 | A | C |
| LR535864.1 | 21082515 | C | T |
| LR535864.1 | 21082565 | C | G |
| LR535864.1 | 21082674 | A | C |
| LR535864.1 | 21082733 | A | C |
| LR535864.1 | 21083077 | G | A |
| LR535864.1 | 21083814 | C | A |
| LR535864.1 | 21084032 | C | T |
| LR535864.1 | 21084277 | G | T |
| LR535864.1 | 21084498 | A | G |
| LR535864.1 | 21084664 | G | A |
| LR535864.1 | 21084704 | T | C |
| LR535864.1 | 21085390 | G | T |
| LR535864.1 | 21085743 | C | T |
| LR535864.1 | 21086074 | T | A |
| LR535864.1 | 21086634 | C | A |
| LR535864.1 | 21086965 | C | T |
| LR535864.1 | 21087074 | A | T |
| LR535864.1 | 21087553 | G | A |
| LR535864.1 | 21087574 | G | C |
| LR535864.1 | 21087877 | G | A |
| LR535864.1 | 21088137 | C | T |
| LR535864.1 | 21088830 | G | T |
| LR535864.1 | 21088923 | A | G |
| LR535864.1 | 21088944 | C | T |
| LR535864.1 | 21089131 | A | C |
| LR535864.1 | 21089629 | C | T |
| LR535864.1 | 21089636 | C | T |
| LR535864.1 | 21093376 | C | T |
| LR535864.1 | 21094238 | A | G |
| LR535864.1 | 21094362 | C | A |
| LR535864.1 | 21094472 | C | A |
| LR535864.1 | 21094779 | C | A |
| LR535864.1 | 21094873 | G | A |
| LR535864.1 | 21095008 | T | A |
| LR535864.1 | 21095346 | C | T |
| LR535864.1 | 21096273 | T | C |
| LR535864.1 | 21097077 | G | A |
| LR535864.1 | 21097098 | T | G |
| LR535864.1 | 21097254 | T | C |
| LR535864.1 | 21097445 | A | G |
| LR535864.1 | 21097493 | C | T |
| LR535864.1 | 21097524 | C | T |
| LR535864.1 | 21097547 | G | A |
| LR535864.1 | 21097778 | C | T |
| LR535864.1 | 21097790 | C | T |
| LR535864.1 | 21098630 | A | G |
| LR535864.1 | 21098909 | A | G |
| LR535864.1 | 21099006 | C | T |
| LR535864.1 | 21099128 | C | G |
| LR535864.1 | 21099235 | T | C |
| LR535864.1 | 21099241 | G | A |
| LR535864.1 | 21099468 | C | T |
| LR535864.1 | 21099490 | C | T |
| LR535864.1 | 21099522 | C | G |
| LR535864.1 | 21099550 | C | T |
| LR535864.1 | 21099810 | A | T |
| LR535864.1 | 21099969 | T | C |
| LR535864.1 | 21100804 | G | A |
| LR535864.1 | 21100946 | A | G |
| LR535864.1 | 21100964 | G | T |
| LR535864.1 | 21101755 | C | T |
| LR535864.1 | 21101769 | G | A |
| LR535864.1 | 21101787 | A | G |
| LR535864.1 | 21101843 | T | C |
| LR535864.1 | 21101942 | T | C |
| LR535864.1 | 21102117 | A | G |
| LR535864.1 | 21102118 | G | T |
| LR535864.1 | 21102121 | G | T |
| LR535864.1 | 21102618 | A | C |
| LR535864.1 | 21102831 | G | A |
| LR535864.1 | 21104388 | G | A |
| LR535864.1 | 21104832 | T | C |
| LR535864.1 | 21105400 | G | A |
| LR535864.1 | 21105453 | G | A |
| LR535864.1 | 21105487 | T | A |
| LR535864.1 | 21105596 | G | A |
| LR535864.1 | 21106048 | G | C |
| LR535864.1 | 21106224 | A | G |
| LR535864.1 | 21106241 | C | T |
| LR535864.1 | 21106242 | C | G |
| LR535864.1 | 21106260 | T | C |
| LR535864.1 | 21106786 | C | T |
| LR535864.1 | 21107618 | G | T |
| LR535864.1 | 21107667 | G | T |
| LR535864.1 | 21107669 | G | C |
| LR535864.1 | 21108590 | C | G |
| LR535864.1 | 21108743 | T | A |
| LR535864.1 | 21108773 | C | T |
| LR535864.1 | 21108778 | A | T |
| LR535864.1 | 21110262 | A | T |
| LR535864.1 | 21110275 | T | G |
| LR535864.1 | 21110323 | C | T |
| LR535864.1 | 21110359 | C | A |
| LR535864.1 | 21111334 | T | C |
| LR535864.1 | 21111378 | C | G |
| LR535864.1 | 21111455 | T | G |
| LR535864.1 | 21112186 | T | C |
| LR535864.1 | 21114078 | G | A |
| LR535864.1 | 21114158 | T | C |
| LR535864.1 | 21114978 | A | T |
| LR535864.1 | 21115969 | G | A |
| LR535864.1 | 21116347 | G | C |
| LR535864.1 | 21116889 | T | C |
| LR535864.1 | 21116906 | C | T |
| LR535864.1 | 21116916 | C | T |
| LR535864.1 | 21117142 | C | A |
| LR535864.1 | 21117143 | C | A |
| LR535864.1 | 21117352 | T | C |
| LR535864.1 | 21117963 | G | A |
| LR535864.1 | 21118178 | A | G |
| LR535864.1 | 21118653 | T | C |
| LR535864.1 | 21118895 | T | C |
| LR535864.1 | 21118999 | A | G |
| LR535864.1 | 21119007 | G | A |
| LR535864.1 | 21119067 | A | G |
| LR535864.1 | 21119227 | T | A |
| LR535864.1 | 21119228 | T | C |
| LR535864.1 | 21119548 | A | T |
| LR535864.1 | 21119880 | C | G |
| LR535864.1 | 21120091 | T | G |
| LR535864.1 | 21120262 | G | A |
| LR535864.1 | 21120341 | T | C |
| LR535864.1 | 21120732 | C | T |
| LR535864.1 | 21120834 | A | G |
| LR535864.1 | 21121330 | G | A |
| LR535864.1 | 21121877 | T | C |
| LR535864.1 | 21122027 | G | T |
| LR535864.1 | 21122094 | C | G |
| LR535864.1 | 21122123 | A | G |
| LR535864.1 | 21122410 | A | G |
| LR535864.1 | 21123012 | C | T |
| LR535864.1 | 21123021 | C | T |
| LR535864.1 | 21123576 | A | C |
| LR535864.1 | 21123639 | C | T |
| LR535864.1 | 21123715 | T | G |
| LR535864.1 | 21123730 | A | G |
| LR535864.1 | 21123847 | C | A |
| LR535864.1 | 21123915 | G | T |
| LR535864.1 | 21124166 | T | A |
| LR535864.1 | 21124178 | A | T |
| LR535864.1 | 21124395 | C | G |
| LR535864.1 | 21124511 | C | G |
| LR535864.1 | 21124713 | G | A |
| LR535864.1 | 21124928 | T | A |
| LR535864.1 | 21125026 | A | G |
| LR535864.1 | 21125088 | G | A |
| LR535864.1 | 21125208 | G | A |
| LR535864.1 | 21125319 | C | T |
| LR535864.1 | 21125326 | A | C |
| LR535864.1 | 21125430 | C | T |
| LR535864.1 | 21125943 | A | G |
| LR535864.1 | 21126046 | C | A |
| LR535864.1 | 21126114 | A | T |
| LR535864.1 | 21126207 | T | G |
| LR535864.1 | 21126446 | T | A |
| LR535864.1 | 21126468 | C | A |
| LR535864.1 | 21126646 | A | T |
| LR535864.1 | 21127511 | A | C |
| LR535864.1 | 21155713 | A | C |
| LR535864.1 | 21156526 | G | A |
| LR535864.1 | 21157433 | G | T |
| LR535864.1 | 21157510 | T | C |
| LR535864.1 | 21157749 | C | A |
| LR535864.1 | 21157750 | A | C |
| LR535864.1 | 21159159 | C | T |
| LR535864.1 | 21159166 | A | T |
| LR535864.1 | 21159197 | A | C |
| LR535864.1 | 21159438 | C | G |
| LR535864.1 | 21159495 | C | T |
| LR535864.1 | 21159555 | G | A |
| LR535864.1 | 21159579 | G | A |
| LR535864.1 | 21160026 | C | T |
| LR535864.1 | 21160111 | G | A |
| LR535864.1 | 21160132 | T | A |
| LR535864.1 | 21160275 | A | G |
| LR535864.1 | 21160452 | T | C |
| LR535864.1 | 21160587 | A | T |
| LR535864.1 | 21160644 | G | C |
| LR535864.1 | 21161182 | A | G |
| LR535864.1 | 21161536 | C | T |
| LR535864.1 | 21161914 | G | A |
| LR535864.1 | 21162161 | G | A |
| LR535864.1 | 21162233 | C | A |
| LR535864.1 | 21162489 | C | A |
| LR535864.1 | 21162583 | G | A |
| LR535864.1 | 21163170 | T | C |
| LR535864.1 | 21163442 | G | T |
| LR535864.1 | 21163549 | C | T |
| LR535864.1 | 21163603 | T | A |
| LR535864.1 | 21163825 | C | A |
| LR535864.1 | 21163979 | C | A |
| LR535864.1 | 21164274 | C | T |
| LR535864.1 | 21164433 | A | C |
| LR535864.1 | 21164456 | G | A |
| LR535864.1 | 21164644 | T | A |
| LR535864.1 | 21164762 | G | T |
| LR535864.1 | 21164776 | A | T |
| LR535864.1 | 21164778 | C | T |
| LR535864.1 | 21164810 | C | A |
| LR535864.1 | 21164927 | G | C |
| LR535864.1 | 21164988 | C | A |
| LR535864.1 | 21165049 | G | A |
| LR535864.1 | 21165188 | T | A |
| LR535864.1 | 21165337 | T | C |
| LR535864.1 | 21165755 | A | G |
| LR535864.1 | 21165785 | T | C |
| LR535864.1 | 21165851 | G | A |
| LR535864.1 | 21165920 | C | T |
| LR535864.1 | 21166152 | T | C |
| LR535864.1 | 21166236 | A | C |
| LR535864.1 | 21166346 | G | T |
| LR535864.1 | 21166578 | C | T |
| LR535864.1 | 21166808 | C | T |
| LR535864.1 | 21166829 | T | C |
| LR535864.1 | 21166964 | A | C |
| LR535864.1 | 21167046 | C | G |
| LR535864.1 | 21167278 | A | G |
| LR535864.1 | 21167352 | A | T |
| LR535864.1 | 21167371 | T | G |
| LR535864.1 | 21167900 | A | G |
| LR535864.1 | 21168128 | T | G |
| LR535864.1 | 21168832 | C | T |
| LR535864.1 | 21170920 | A | G |
| LR535864.1 | 21172277 | C | T |
| LR535864.1 | 21172278 | A | G |
| LR535864.1 | 21172320 | T | C |
| LR535864.1 | 21172749 | C | G |
| LR535864.1 | 21173894 | C | T |
| LR535864.1 | 21173918 | G | A |
| LR535864.1 | 21174128 | G | A |
| LR535864.1 | 21174316 | G | A |
| LR535864.1 | 21174420 | G | A |
| LR535864.1 | 21174586 | T | G |
| LR535864.1 | 21175083 | A | C |
| LR535864.1 | 21175791 | T | G |
| LR535864.1 | 21175870 | A | T |
| LR535864.1 | 21176129 | G | T |
| LR535864.1 | 21176133 | T | G |
| LR535864.1 | 21176188 | A | C |
| LR535864.1 | 21176189 | T | A |
| LR535864.1 | 21176434 | G | T |
| LR535864.1 | 21177016 | A | C |
| LR535864.1 | 21177040 | T | C |
| LR535864.1 | 21177062 | A | G |
| LR535864.1 | 21177516 | G | A |
| LR535864.1 | 21177828 | T | C |
| LR535864.1 | 21178118 | G | A |
| LR535864.1 | 21178119 | T | A |
| LR535864.1 | 21178431 | T | C |
| LR535864.1 | 21178596 | A | T |
| LR535864.1 | 21178882 | C | T |
| LR535864.1 | 21178941 | A | C |
| LR535864.1 | 21178983 | G | A |
| LR535864.1 | 21179242 | G | A |
| LR535864.1 | 21179447 | A | C |
| LR535864.1 | 21179461 | A | G |
| LR535864.1 | 21179876 | C | T |
| LR535864.1 | 21180008 | A | G |
| LR535864.1 | 21180012 | T | A |
| LR535864.1 | 21180191 | C | T |
| LR535864.1 | 21180197 | T | C |
| LR535864.1 | 21180202 | T | C |
| LR535864.1 | 21180349 | G | A |
| LR535864.1 | 21180586 | C | T |
| LR535864.1 | 21180635 | C | T |
| LR535864.1 | 21180831 | A | G |
| LR535864.1 | 21181012 | A | C |
| LR535864.1 | 21181382 | A | G |
| LR535864.1 | 21181393 | C | T |
| LR535864.1 | 21181776 | A | G |
| LR535864.1 | 21182234 | G | T |
| LR535864.1 | 21183792 | C | T |
| LR535864.1 | 21183891 | C | T |
| LR535864.1 | 21329246 | G | T |
| LR535864.1 | 21329452 | G | A |
| LR535864.1 | 21329967 | A | C |
| LR535864.1 | 21330074 | G | T |
| LR535864.1 | 21332922 | T | C |
| LR535864.1 | 21333979 | A | G |
| LR535864.1 | 21341110 | A | G |
| LR535864.1 | 21341302 | A | C |
| LR535864.1 | 21342575 | G | C |
| LR535864.1 | 21343353 | C | A |
| LR535864.1 | 21343355 | T | G |
| LR535864.1 | 21343633 | A | C |
| LR535864.1 | 21343776 | G | A |
| LR535864.1 | 21343817 | T | A |
| LR535864.1 | 21344031 | C | A |
| LR535864.1 | 21344311 | T | A |
| LR535864.1 | 21344349 | C | G |
| LR535864.1 | 21344549 | G | A |
| LR535864.1 | 21344656 | C | T |
| LR535864.1 | 21344808 | A | G |
| LR535864.1 | 21344858 | T | A |
| LR535864.1 | 21344906 | C | T |
| LR535864.1 | 21345056 | C | T |
| LR535864.1 | 21345090 | A | T |
| LR535864.1 | 21356566 | A | C |
| LR535864.1 | 22252797 | A | T |
| LR535864.1 | 22252833 | G | T |
| LR535864.1 | 22252880 | A | G |
| LR535864.1 | 22252902 | G | A |
| LR535864.1 | 22253375 | T | A |
| LR535864.1 | 22253428 | A | T |
| LR535864.1 | 22255111 | C | G |
| LR535864.1 | 22255389 | T | C |
| LR535864.1 | 22257696 | C | T |
| LR535864.1 | 22258355 | C | T |
| LR535864.1 | 22258367 | C | T |
| LR535864.1 | 22259270 | A | G |
| LR535864.1 | 22259278 | G | A |
| LR535864.1 | 22259328 | A | G |
| LR535864.1 | 22259331 | C | A |
| LR535864.1 | 22259384 | G | C |
| LR535864.1 | 22259420 | G | A |
| LR535864.1 | 22259562 | C | A |
| LR535864.1 | 22259571 | C | T |
| LR535864.1 | 22259581 | T | G |
| LR535864.1 | 22259595 | G | A |
| LR535864.1 | 22261698 | G | A |
| LR535864.1 | 22262210 | A | T |
| LR535864.1 | 22262221 | C | T |
| LR535864.1 | 22262239 | C | T |
| LR535864.1 | 22262265 | T | G |
| LR535864.1 | 22262269 | T | A |
| LR535864.1 | 22262314 | T | A |
| LR535864.1 | 22262327 | G | A |
| LR535864.1 | 22265336 | C | T |
| LR535864.1 | 22267091 | G | A |
| LR535864.1 | 22267273 | T | G |
| LR535864.1 | 22267373 | A | C |
| LR535864.1 | 22268264 | G | T |
| LR535864.1 | 22268749 | C | A |
| LR535864.1 | 22268753 | G | A |
| LR535864.1 | 22268779 | G | C |
| LR535877.1 | 17047390 | A | T |
| LR535877.1 | 17047422 | G | T |
| LR535877.1 | 17047423 | A | G |
| LR535877.1 | 17047434 | G | A |
| LR535877.1 | 17047435 | A | C |
| LR535877.1 | 17047446 | G | A |
| LR535877.1 | 17048185 | T | C |
| LR535877.1 | 17048205 | A | C |
| LR535877.1 | 17048218 | G | A |
| LR535877.1 | 17048222 | A | G |
| LR535877.1 | 17048225 | T | A |
| LR535877.1 | 17048239 | A | T |
| LR535877.1 | 17048260 | C | T |
| LR535877.1 | 17048261 | G | T |
| LR535877.1 | 17048263 | C | A |
| LR535877.1 | 17048874 | C | T |
| LR535877.1 | 17048877 | G | C |
| LR535877.1 | 17049306 | G | A |
| LR535877.1 | 17049310 | C | T |
| LR535877.1 | 17049314 | G | T |
| LR535877.1 | 17049450 | G | A |
| LR535877.1 | 17049451 | A | C |
| LR535877.1 | 17049466 | A | T |
| LR535877.1 | 17049468 | G | T |
| LR535877.1 | 17049504 | A | G |
| LR535877.1 | 17049883 | T | A |
| LR535877.1 | 17051213 | T | G |
| LR535877.1 | 17051221 | C | A |
| LR535877.1 | 17051451 | C | T |
| LR535877.1 | 17051462 | G | T |
| LR535877.1 | 17051469 | T | A |
| LR535877.1 | 17051775 | G | T |
| LR535877.1 | 17054966 | G | A |
| LR535877.1 | 17055118 | C | T |
| LR535877.1 | 17055130 | G | T |
| LR535877.1 | 17055156 | A | T |
| LR535877.1 | 17055157 | C | A |
| LR535877.1 | 17055169 | T | A |
| LR535877.1 | 17055173 | C | A |
| LR535877.1 | 17055174 | G | A |
| LR535877.1 | 17055230 | G | A |

**Supplementary Table S3. The experimental data for the proportion of homozygous (*P_hom_*) males and females versus read coverage (*x*).** The 95% confidence limits (*c_l_, c_h_*) are obtained from the exact binomial test using the number of homozygous genotypes (*N_hom_*), the total number (*n*) and the expected proportion of homozygous genotypes under the null hypothesis (*P_hom_(H_0_*)) of the theoretical model (Chenuil, 2012): assuming the females are homozygous $P(x|hom)=1$ and the males are heterozygous $P(x|het)={1/2}^{x-1}$.

| ***sex*** | ***x*** | ***P_hom_*** | ***c_l_*** | ***c_h_*** | ***test*** | ***N_hom_*** | ***n*** | ***P_hom_(H_0_)*** | ***alternat.*** | ***p*-value** | ***H_0_*rejected** |
| --- | --- | --- | --- | --- | --- | --- | --- | --- | --- | --- | --- |
| F | 1 | 1.000 | 0.999 | 1.000 | Exact Binomial | 4154 | 4154 | 1.000000 | two.sided | 1.00e+00 | FALSE |
| F | 2 | 0.997 | 0.995 | 0.998 | Exact Binomial | 4404 | 4417 | 1.000000 | two.sided | 0.00e+00 | TRUE |
| F | 3 | 0.992 | 0.988 | 0.995 | Exact Binomial | 3523 | 3552 | 1.000000 | two.sided | 0.00e+00 | TRUE |
| F | 4 | 0.994 | 0.991 | 0.997 | Exact Binomial | 2850 | 2866 | 1.000000 | two.sided | 0.00e+00 | TRUE |
| F | 5 | 0.992 | 0.987 | 0.995 | Exact Binomial | 1993 | 2009 | 1.000000 | two.sided | 0.00e+00 | TRUE |
| F | 6 | 0.996 | 0.991 | 0.999 | Exact Binomial | 1493 | 1499 | 1.000000 | two.sided | 0.00e+00 | TRUE |
| F | 7 | 0.993 | 0.985 | 0.997 | Exact Binomial | 981 | 988 | 1.000000 | two.sided | 0.00e+00 | TRUE |
| F | 8 | 0.993 | 0.983 | 0.998 | Exact Binomial | 679 | 684 | 1.000000 | two.sided | 0.00e+00 | TRUE |
| F | 9 | 1.000 | 0.991 | 1.000 | Exact Binomial | 429 | 429 | 1.000000 | two.sided | 1.00e+00 | FALSE |
| F | 10 | 0.990 | 0.971 | 0.998 | Exact Binomial | 292 | 295 | 1.000000 | two.sided | 0.00e+00 | TRUE |
| F | 11 | 0.979 | 0.947 | 0.994 | Exact Binomial | 187 | 191 | 1.000000 | two.sided | 0.00e+00 | TRUE |
| F | 12 | 0.986 | 0.950 | 0.998 | Exact Binomial | 139 | 141 | 1.000000 | two.sided | 0.00e+00 | TRUE |
| F | 13 | 0.939 | 0.852 | 0.983 | Exact Binomial | 62 | 66 | 1.000000 | two.sided | 0.00e+00 | TRUE |
| F | 14 | 1.000 | 0.935 | 1.000 | Exact Binomial | 55 | 55 | 1.000000 | two.sided | 1.00e+00 | FALSE |
| F | 15 | 1.000 | 0.815 | 1.000 | Exact Binomial | 18 | 18 | 1.000000 | two.sided | 1.00e+00 | FALSE |
| F | 16 | 0.950 | 0.751 | 0.999 | Exact Binomial | 19 | 20 | 1.000000 | two.sided | 0.00e+00 | TRUE |
| F | 17 | 1.000 | 0.735 | 1.000 | Exact Binomial | 12 | 12 | 1.000000 | two.sided | 1.00e+00 | FALSE |
| F | 18 | 1.000 | 0.590 | 1.000 | Exact Binomial | 7 | 7 | 1.000000 | two.sided | 1.00e+00 | FALSE |
| F | 19 | 1.000 | 0.158 | 1.000 | Exact Binomial | 2 | 2 | 1.000000 | two.sided | 1.00e+00 | FALSE |
| F | 20 | 1.000 | 0.158 | 1.000 | Exact Binomial | 2 | 2 | 1.000000 | two.sided | 1.00e+00 | FALSE |
| F | 21 | 1.000 | 0.398 | 1.000 | Exact Binomial | 4 | 4 | 1.000000 | two.sided | 1.00e+00 | FALSE |
| M | 1 | 0.992 | 0.989 | 0.994 | Exact Binomial | 4312 | 4348^†^ | 1.000000 | two.sided | 0.00e+00 | TRUE |
| M | 2 | 0.646 | 0.632 | 0.659 | Exact Binomial | 3155 | 4886 | 0.500000 | two.sided | 0.00e+00 | TRUE |
| M | 3 | 0.396 | 0.381 | 0.410 | Exact Binomial | 1684 | 4257 | 0.250000 | two.sided | 0.00e+00 | TRUE |
| M | 4 | 0.251 | 0.237 | 0.266 | Exact Binomial | 864 | 3438 | 0.125000 | two.sided | 0.00e+00 | TRUE |
| M | 5 | 0.162 | 0.148 | 0.177 | Exact Binomial | 428 | 2638 | 0.062500 | two.sided | 0.00e+00 | TRUE |
| M | 6 | 0.123 | 0.109 | 0.138 | Exact Binomial | 254 | 2065 | 0.031250 | two.sided | 0.00e+00 | TRUE |
| M | 7 | 0.089 | 0.074 | 0.105 | Exact Binomial | 125 | 1407 | 0.015625 | two.sided | 0.00e+00 | TRUE |
| M | 8 | 0.088 | 0.070 | 0.108 | Exact Binomial | 77 | 878 | 0.007812 | two.sided | 0.00e+00 | TRUE |
| M | 9 | 0.057 | 0.040 | 0.078 | Exact Binomial | 35 | 614 | 0.003906 | two.sided | 0.00e+00 | TRUE |
| M | 10 | 0.068 | 0.044 | 0.098 | Exact Binomial | 25 | 370 | 0.001953 | two.sided | 0.00e+00 | TRUE |
| M | 11 | 0.045 | 0.023 | 0.077 | Exact Binomial | 12 | 267 | 0.000977 | two.sided | 0.00e+00 | TRUE |
| M | 12 | 0.067 | 0.036 | 0.111 | Exact Binomial | 13 | 195 | 0.000488 | two.sided | 0.00e+00 | TRUE |
| M | 13 | 0.045 | 0.015 | 0.102 | Exact Binomial | 5 | 111 | 0.000244 | two.sided | 1.00e-10 | TRUE |
| M | 14 | 0.083 | 0.028 | 0.184 | Exact Binomial | 5 | 60 | 0.000122 | two.sided | 0.00e+00 | TRUE |
| M | 15 | 0.140 | 0.053 | 0.279 | Exact Binomial | 6 | 43 | 0.000061 | two.sided | 0.00e+00 | TRUE |
| M | 16 | 0.091 | 0.019 | 0.243 | Exact Binomial | 3 | 33 | 0.000031 | two.sided | 2.00e-10 | TRUE |
| M | 17 | 0.000 | 0.000 | 0.168 | Exact Binomial | 0 | 20 | 0.000015 | two.sided | 1.00e+00 | FALSE |
| M | 18 | 0.091 | 0.011 | 0.292 | Exact Binomial | 2 | 22 | 0.000008 | two.sided | 1.34e-08 | TRUE |
| M | 19 | 0.000 | 0.000 | 0.336 | Exact Binomial | 0 | 9 | 0.000004 | two.sided | 1.00e+00 | FALSE |
| M | 20 | 0.000 | 0.000 | 0.308 | Exact Binomial | 0 | 10 | 0.000002 | two.sided | 1.00e+00 | FALSE |
| M | 21 | 0.000 | 0.000 | 0.410 | Exact Binomial | 0 | 7 | 0.000001 | two.sided | 1.00e+00 | FALSE |

^†^ The variant caller FreeBayes was used in this study. This is a haplotype based variant caller that uses the haplotype information from all individuals in the file to make calls (Garrison and Marth, 2012). Therefore, a few heterozygous genotypes have been called even though the coverage was 1.
